# Analysis of the correlation between morphological classification of the middle glenohumeral ligament and subscapularis tears

**DOI:** 10.1038/s41598-025-11205-5

**Published:** 2025-07-13

**Authors:** Tao Liu, Tao Meng, Zhen Huang, Yaning Wang, Hui Shi, Linwei Wang, Chao Lin

**Affiliations:** 1 Department of Bone and Joint Surgery and Sports Medicine, Binzhou Medical University Hospital, Binzhou, 256603 Shandong China; 2 Department of Nephrology, Binzhou Medical University Hospital, Binzhou, China

**Keywords:** Shoulder arthroscopy, Rotator cuff tear, Middle glenohumeral ligament, Subscapularis tears, Musculoskeletal system, Orthopaedics

## Abstract

The purpose of this study was to evaluate the correlation between the morphological classification of the middle glenohumeral ligament (MGHL) and subscapularis tears and to evaluate whether surgical release of the MGHL is warranted in patients with subscapularis tears. A retrospective analysis was performed on the surgical videos of shoulder arthroscopic rotator cuff repair surgeries conducted by the same surgeon from September 2020 to September 2021. The MGHL was classified morphologically into two types: overall classification and lateral insertion classification, and the occurrence of subscapularis tears was recorded for each type. The chi-square test was used to analyze the differences in the number of subscapularis tears among the classification groups. Out of 122 patients, 44 (36.07%) were male and 78 (63.93%) were female, with an average age of 55.03 ± 7.35 years. According to the overall classification of MGHL, there were 54 cases of Type I (44.26%), 32 cases of Type II (26.23%), 28 cases of Type III (22.95%), 2 cases of Type IV (1.64%), and 6 cases of Type V (4.92%). For the lateral insertion classification of MGHL, there were 53 cases of Type A (43.44%) and 69 cases of Type B (69%). There was no statistically significant difference in the number of subscapularis tears among the classification groups . This retrospective study found no correlation between the morphological classification of MGHL and subscapularis tears. For patients with rotator cuff tears who do not have frozen shoulder, caution should be exercised when performing release of the MGHL.

## Introduction

The middle glenohumeral ligament (MGHL) is a component of the lateral aspect of the rotator interval, maintaining the anterior stability of the shoulder joint along with the superior and inferior glenohumeral ligaments. It exhibits various morphological variations. It is generally believed to originate from the anatomic neck of the scapula or the anterior labrum and insert on the lesser tuberosity of the humerus, passing obliquely over the superior glenohumeral ligament and the coracohumeral ligament. However, recent studies have found that the MGHL inserts on the articular surface of the subscapularis muscle^1,2^. Some clinical literature^3^ has suggested that the MGHL can cause wear of the subscapularis, leading to complete subscapularis tendon injuries (SAM injuries) and recommends the release of the MGHL during surgery. However, the relationship between different morphologies of the MGHL and subscapularis muscle injury and the necessity of releasing the MGHL have not been well studied.

The purpose of this study is to analyze the correlation between the morphological classification of the MGHL and subscapularis muscle injury and to explore the necessity of releasing the MGHL during surgery.

## Materials and methods

A retrospective study was conducted on 122 cases of arthroscopic rotator cuff repair surgery videos performed by the same surgeon from September 2020 to September 2021, based on inclusion and exclusion criteria.

### Ethical approval statement

This study was approved by the Research Ethics Committee of BinZhou Medical University Hospital in BinCheng, BinZhou, PR China (IRB No. 2023-LW-155), All procedures conducted in this study adhered to the ethical standards set by the university’s committee and complied with the 1964 Declaration of Helsinki and its subsequent amendments. Informed consent was obtained from all participants prior to inclusion in the analysis.

Inclusion criteria:

- Diagnosis: rotator cuff tear.
- No age limit.

Exclusion criteria:

- Previous shoulder arthroscopy surgery.
- Incomplete surgical video.
- Shoulder joint instability.
- Bankart lesion.
- Frozen shoulder.
- With manual release or arthroscopic release during surgery.

**Fig. 1:**
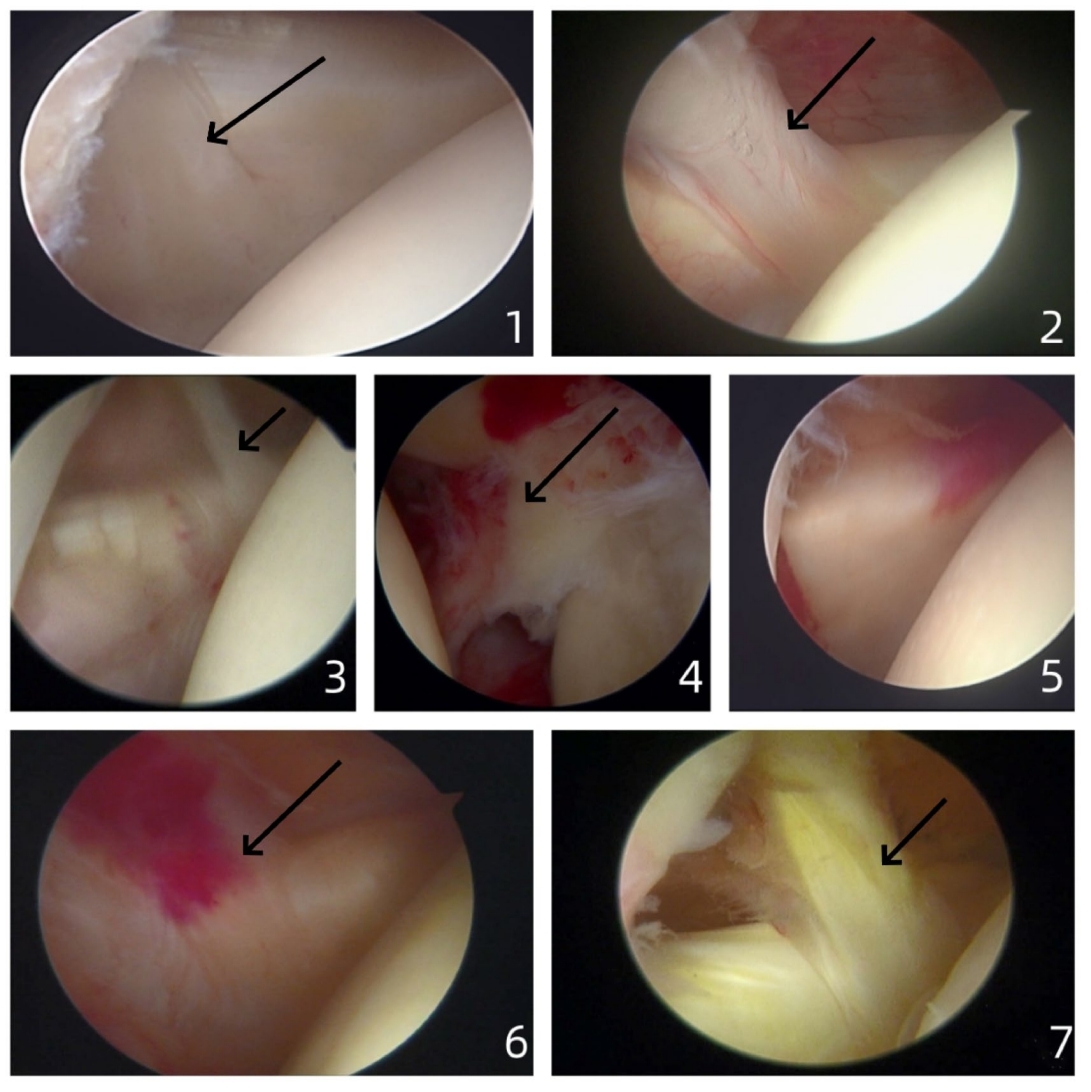
1: Flat: The medial edge of the MGHL can only be seen without rotating the humeral head, thick (a probe placed behind it cannot be seen); 2: Cord-like: Both the medial and lateral edges of the MGHL are visible; 3: Hypoplastic: It appears as a strand or membrane, thin (a probe placed behind it can be seen); 4: Buford complex; 5: Absent: 6: Type A, The insertion of the MGHL is close to the labrum; 7: Type B, The insertion of the MGHL is close to the humeral head: The insertion of the MGHL is close to the posterior humeral head and can only be identified after rotating the humeral head.

The MGHL varies greatly among individuals, with various morphological classifications^4–6^. This study adopted the arthroscopic overall classification (viewing approach: 1.5–3 cm below the posterior-lateral end of the acromion, 1 cm medial; 30° arthroscope) and the lateral insertion classification (Fig. 1). After carefully reviewing the surgical videos, three senior physicians collectively identified five types. The point of contention was whether Type 3 and Type 5 should be categorized as one class. Following discussions, it was unanimously agreed to classify them into two distinct types, with the classification criterion being whether the probe placed behind them could be clearly visualized. Overall classification of MGHL: Types I–V: Type I Flat: The medial edge of the MGHL can only be seen without rotating the humeral head, thick (a probe placed behind it cannot be seen). Type II Cord-like: Both the medial and lateral edges of the MGHL are visible. Type III Hypoplastic: It appears as a strand or membrane, thin (a probe placed behind it can be seen). Type IV Buford complex^7^. Type V Absent. Lateral insertion classification of MGHL: Type A: The insertion of the MGHL is close to the labrum; Type B: The insertion of the MGHL is close to the humeral head: The insertion of the MGHL is close to the posterior humeral head and can only be identified after rotating the humeral head.

Three senior physicians (with the title of Deputy Chief Physician or above, with more than 100 shoulder arthroscopies/year, Tao Liu, Tao Meng, Chao Lin) watched the surgical videos separately and made records. If there were different opinions, the type was decided after discussion.

All patients underwent general anesthesia combined with brachial plexus nerve block anesthesia, in a lateral decubitus position, with the affected upper limb abducted at 45° and flexed at 15–20° for lateral traction, with a traction weight of 5 kg. The perfusion pump maintained a perfusion pressure of 30 mmHg, and the perfusion fluid was 0.9% sodium chloride solution with added epinephrine (1 mg/L epinephrine)^8^.

SPSS 19.0 statistical software was used for data analysis. Qualitative data were represented as cases (%), and the chi-square test was used for group comparisons. Quantitative data were represented as (mean ± standard deviation). *P* < 0.05 was considered statistically significant.

## Results

This study included 122 patients, of whom 44 were male (36.07%) and 78 were female (63.93%), with an average age of 55.03 ± 7.35 years. Among the 122 shoulder arthroscopy surgeries, the overall classification of MGHL was: Type I in 54 cases (44.26%), Type II in 32 cases (26.23%), Type III in 28 cases (22.95%), Type IV in 2 cases (1.64%), and Type V in 6 cases (4.92%). The lateral insertion classification of MGHL was: Type A in 53 cases (43.44%) and Type B in 69 cases (69%). The condition of subscapularis tears in each type of overall classification of MGHL is shown in Table 1, with no statistically significant difference in the difference of subscapularis tears among the groups (*p* = 0.089); the condition of subscapularis tears in each type of lateral insertion classification of MGHL is shown in Table 2, with no statistically significant difference in the difference of subscapularis tears among the groups (*p* = 0.22).

**Table 1:** Overall classification of MGHL and subscapularis tears (Sbt).

Classification	Sbt Yes (*n*)	Sbt No (*n*)	Total rate (each classifcation)	*P*
I	32	22	44.26%	
II	25	7	26.23%	
III	23	5	22.95%	
IV	2	0	1.64%	
V	3	3	4.92%	0.089

**Table 2:** Classification of MGHL lateral insertion and subscapularis tears (Sbt).

Classification	Sbt Yes (*n*)	Sbt No (*n*)	Total rate (each classifcation)	*P*
A	40	13	43.44%	
B	45	24	56.56%	0.22

## Discussion

In this study, the classification criteria for MGHL morphology were not entirely consistent with previous studies, among which only the overall classification of Type IV and V and the lateral insertion classification were comparable with previous research. The proportion of Type IV MGHL was 1.64%, which is within the range of 1.2−7.5% reported in previous studies^7^. The proportion of Type V MGHL was 4.92%, lower than the 63% reported by Steinbeck et al. based on 104 cadaver specimens, and higher than the 2% conclusion drawn by Collotte et al. based on 107 arthroscopic shoulder cases. The reasons for this result may include: (1) Arthroscopic technology can more subtly observe the structure;^4,5,9^ (2) In patients with frozen shoulder, the hypoplastic MGHL adheres to other structures within the rotator interval, making it indistinguishable^10^. In this study, the proportion of Type A MGHL insertion was 43.44%, and Type B was 56.56%, which is similar to the results of Collotte et al.^5^.

With the development of arthroscopic technology, more and more scholars^4–6,11^ believe that the lateral insertion of the MGHL is on the articular surface of the subscapularis muscle and can retract medially with the injured subscapularis muscle, which can explain the clinical significance of the MGHL in subscapularis muscle injury: Hsu KL^12^ et al. found that the preoperative magnetic resonance imaging of the displaced MGHL is correlated with the degree of subscapularis muscle injury, and a retraction ratio > 1.25 can predict severe subscapularis muscle injury. Laurent et al.^13^ believe that the position of the MGHL after reduction can be used to judge the injury and post-repair reduction condition of the subscapularis muscle. Brady^3^ et al. believe that an excessively thick MGHL and an insertion point too close to the subscapularis muscle insertion can cause wear of the articular surface of the subscapularis muscle, and is called SAM injury, recommending the release of the MGHL during surgery^14^. However, in this study, there was no statistically significant difference in subscapularis muscle injury among different types of MGHL insertion points, which may be due to: 1) The sample size of this study is too small. “While our sample size (n = 122) is comparable to prior morphological studies [12], the uneven distribution of MGHL subtypes (e.g., only 2 Type IV cases) may limit subgroup analyses. Future multicenter studies with larger cohorts are needed to validate these preliminary findings.“2) There are various high-risk factors for subscapularis muscle injury: age, diabetes, and acromial morphology, etc^15,16^. and this study did not analyze the combined effect of MGHL and these factors. 3) This study, like previous studies, found^17^ that when the affected limb is abducted more than 60° and externally rotated, the MGHL rubs against the subscapularis muscle, so it cannot be determined whether SAM injury is related to long-term engagement in certain activities in daily life. 4) The role of coracoid process morphology in subscapularis muscle injury is controversial^18,19^, because the subscapularis muscle is located between the coracoid process and the MGHL, and the positional relationship between the coracoid process and MGHL may have an impact on subscapularis muscle injury, but this study did not involve it.

The MGHL statically restricts the rotational movement of the humeral head during shoulder adduction and also restricts the downward translation movement of the humeral head during adduction and external rotation of the shoulder. In addition, the MGHL also restricts the anterior and posterior translation movement of the arm during abduction (45°) and external rotation, and these restrictions are more important when there is a Buford complex. However, Hagiwara Y et al. found that releasing the MGHL in patients with frozen shoulder can increase the range of joint motion without affecting shoulder joint stability^1,14,20,21^. Therefore, caution should be exercised when performing release of the MGHL in patients without frozen shoulder, so as not to affect the stability of the shoulder joint.

## Conclusion

Through this retrospective study, it was found that there is no correlation between the morphological classification of the MGHL and subscapularis muscle injury; for patients with rotator cuff injury without frozen shoulder, caution should be exercised when performing release of the MGHL.

## Data Availability

The datasets used and/or analysed during the current study available from the corresponding author on reasonable request.
